# When Insufflation Goes Awry: Massive Gas Embolism During Laparoscopic Surgery

**DOI:** 10.1016/j.case.2022.12.001

**Published:** 2023-01-18

**Authors:** Liam Kennedy, Raffael Pereira Cezar Zamper

**Affiliations:** a Schulich School of Medicine and Dentistry, University of Western Ontario, London, Ontario, Canada; b Department of Anaesthesia and Perioperative Medicine, Schulich School of Medicine and Dentistry, University of Western Ontario, London, Ontario, Canada

**Keywords:** Carbon dioxide embolism, Transesophageal echocardiography, Pneumoperitoneum, Cardiovascular collapse

## Abstract

• CO_2_ embolism is a rare but life-threatening complication of laparoscopic surgery.
• CO_2_ embolism presents as cardiorespiratory failure requiring prompt intervention.
• The gold standard for diagnostic investigation is TEE.
• Treatment includes desufflation, high FiO_2_, and cardiopulmonary resuscitation.
• Systemic embolization is the most-feared complication of CO_2_ embolism.

## Graphical abstract

**Figure:**
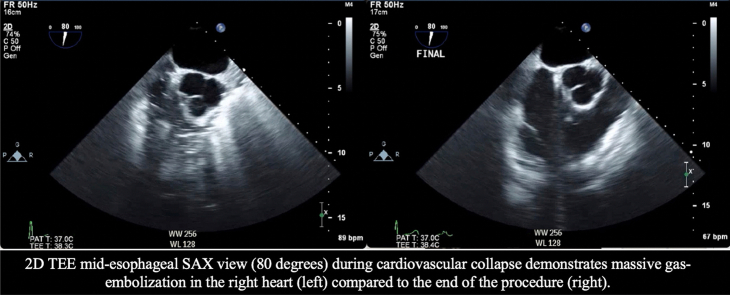

## Introduction

Since their induction into routine clinical practice, laparoscopic techniques have revolutionized the field of general surgery. Minimally invasive techniques yield fewer postoperative complications, shortened hospital stays, and reduced patient recovery times.^1^ However, there are significant risks involved in the use of CO_2_ pneumoperitoneum insufflation. These commonly include hypotension, hypertension, arrythmias, and pulmonary barotrauma.^2^ More serious complications such as CO_2_ embolism are far less frequent, occurring in only 0.001^3^ to 0.59%^4^ of all laparoscopies, but are associated with a much higher mortality rate of 28%.^5^

We present a patient with cardiovascular collapse of unknown origin during a laparoscopic liver resection that was found to be caused by a massive CO_2_ embolism, diagnosed via emergent transesophageal echocardiogram (TEE), and resolved with minimal complications following conversion to open surgery.

## Case Presentation

**Figure 1:**
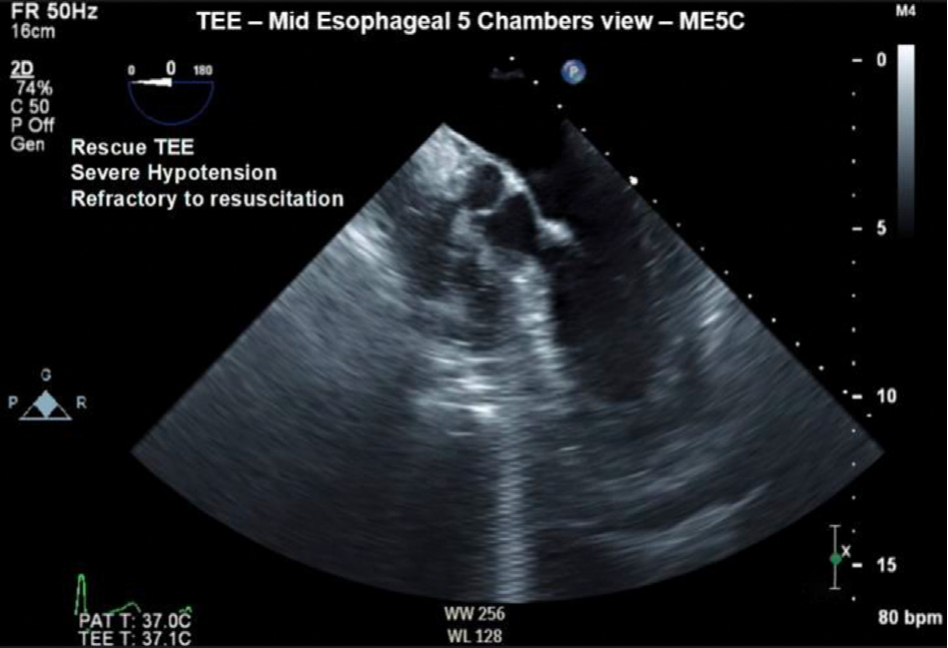
Two-dimensional TEE, midesophageal 5-chamber view (0°), diastolic phase, demonstrates a significant amount of echo-bright densities in the right heart.

**Figure 2:**
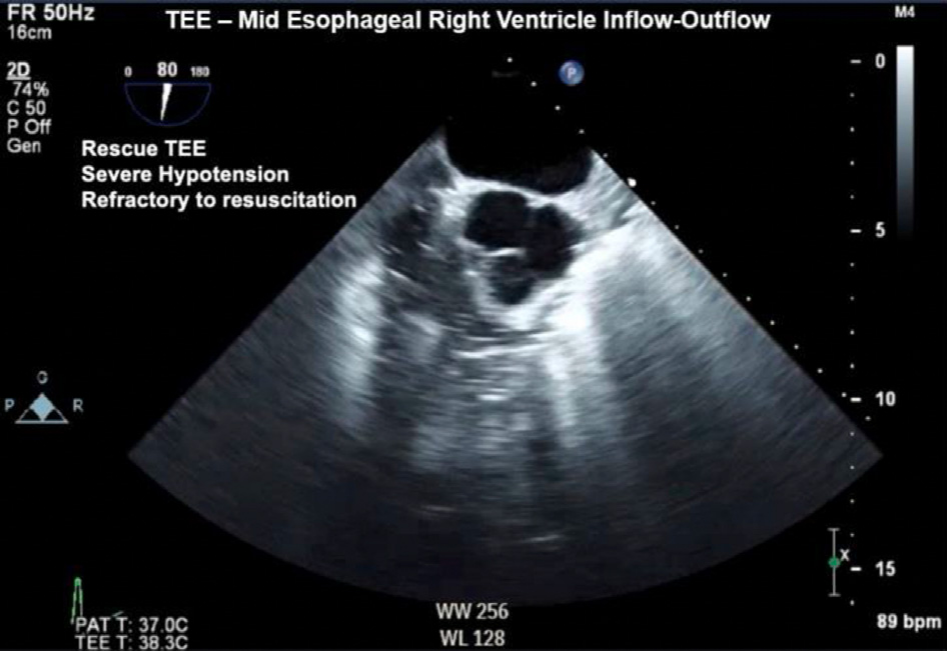
Two-dimensional TEE, midesophageal short-axis view (80°), diastolic phase, demonstrates the right ventricular inflow-outflow display with a significant amount of echo-bright densities in the right heart.

A 54-year-old man was scheduled to undergo a laparoscopic liver resection for a right sectoral intrahepatic cholangiocarcinoma, post–neoadjuvant chemotherapy. Medical history was significant for hemochromatosis, hypertension, gastroesophageal reflux disease, dyslipidemia, thyroid nodule, and a 25-pack-year smoking history. Preanesthetic assessment was unremarkable, and the patient was equipped with a 5-lead electrocardiogram, pulse oximeter, and noninvasive blood pressure cuff upon arrival in the operating room. After induction, a second large-bore peripheral intravenous and arterial line were placed without issue. Mechanical ventilation was initiated uneventfully; however, the capnography almost immediately displayed a pattern of mild obstruction, with a flattening of the expiratory upstroke and subsequent steepening of the alveolar plateau resembling the typical “shark-fin” appearance. These changes were thought to be reflective of hyperreactive airways secondary to underlying COPD given the patient’s significant smoking history. As the patient was still ventilating adequately, no intervention was undertaken at this time. After installation of pneumoperitoneum, spO_2_ dropped to the low 90s and an arterial blood gas (ABG) was drawn, which illustrated a mild respiratory acidosis (pH = 7.33, pCO_2_ = 59). Subsequent auscultation of the lungs revealed generalized wheezing. Given the patient’s history of smoking and intraoperative findings compatible with underlying chronic obstructive pulmonary disease, multiple interventions were performed to treat bronchospasm as the cause of poor blood oxygenation, without success. Additionally, endotracheal tube placement was verified to rule out the possibility of iatrogenic single-lung ventilation secondary to a cephalad diaphragmatic displacement following insufflation. When these interventions failed, empiric treatment with epinephrine was initiated for potential anaphylaxis. Despite this, the patient continued to deteriorate over the subsequent hour, and we eventually discarded our previous hypotheses once the patient became persistently hypotensive, despite increasing vasopressor use, thus signaling significant cardiovascular decompensation. A repeat ABG was done, which showed a now near-critical respiratory acidosis with decreasing oxygenation (pH = 7.22, pCO_2_ of 71, CO_2_ gap of 32 [ETCO_2_ = 39], and pO_2_ = 89). Given the significant V/Q mismatch present on the ABG, we began to suspect shunting and called for an emergent TEE to assess. Shortly thereafter, although surgery was uneventful and without any significant blood loss, the patient became profoundly hypoxemic (SpO_2_ = 75%) and severely hypotensive (systolic blood pressure = 60, diastolic blood pressure = 40), refractory to the use of vasopressors. Emergent TEE was performed, demonstrating significant gas embolism in the right heart, including the right atrium, right ventricle, and proximal pulmonary artery (Figures 1 and 2, Videos 1 and 2).

**Figure 3:**
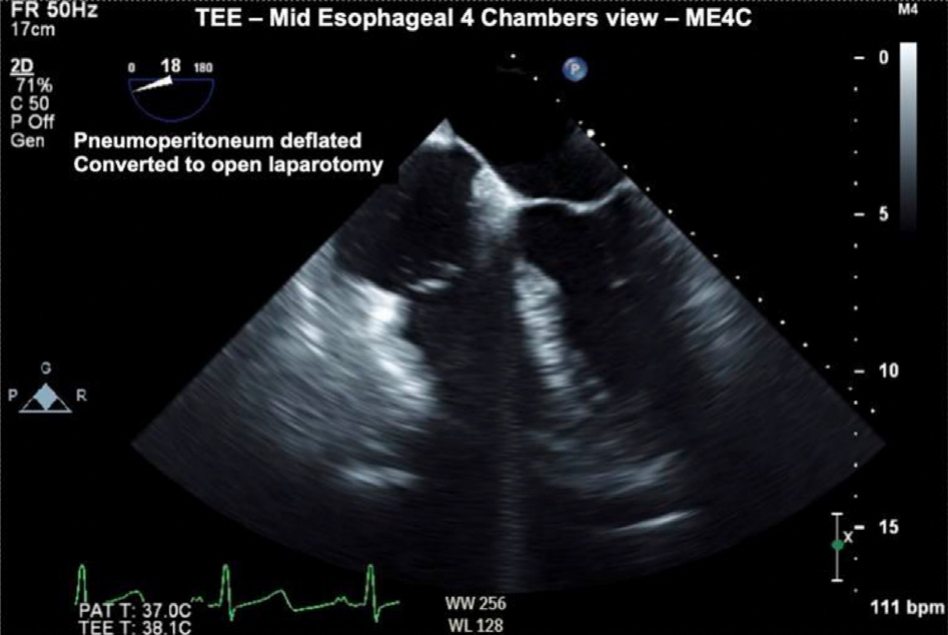
Two-dimensional TEE, midesophageal 4-chamber view (18°), systolic phase approximately 5 minutes postconversion from laparoscopy to laparotomy, demonstrates a few remaining echo-bright densities in the right heart with adequate left ventricular filling and resolution of the previously noted leftward deviation of the atrial septum suggesting normalization of previously elevated RA pressures.

**Figure 4:**
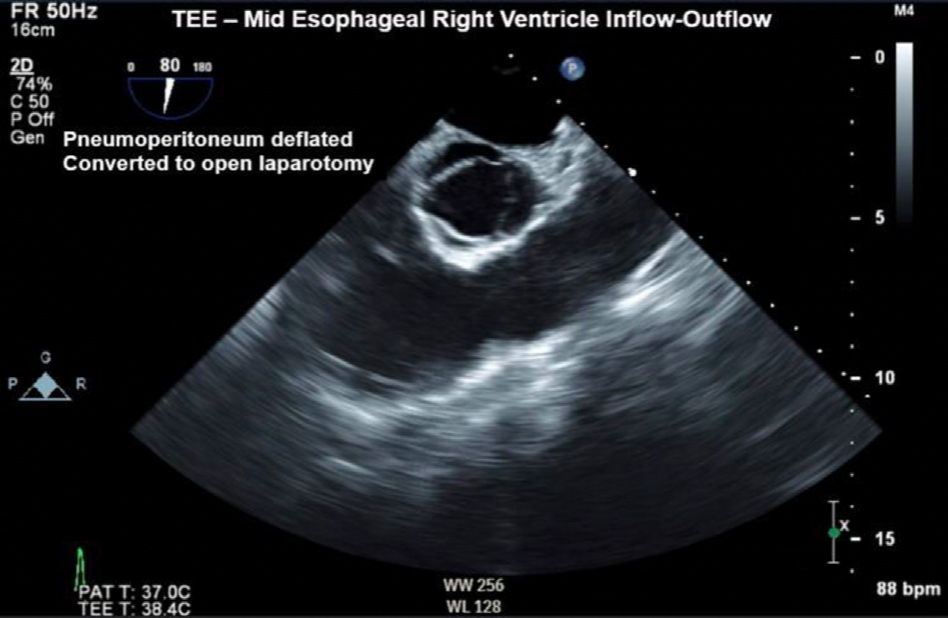
Two-dimensional TEE, midesophageal short-axis view (80°), systolic phase, demonstrates the right ventricular inflow-outflow display approximately 5 minutes postconversion to laparotomy with minimal amounts of echo-bright densities remaining in the right heart.

The exact etiology for this was unclear, as decompensation began prior to liver resection and without any visibly opened vasculature that could be amenable to aspirating large volumes of CO_2_. However, the surgical team did note significant collateral circulation in the abdominal wall, compatible with the finding of a cirrhotic liver, and thus hypothesized that the embolism was likely secondary to injury of one of these collateral vessels during port insertion. The injured vessel itself was never directly visualized, and no active bleeding was identified. Importantly, no other iatrogenic cause was readily identifiable from the anesthesia perspective. A central venous line was not placed at the initiation of the case, and there was no evidence present to support embolization from a pressurized intravenous bag or peripheral line. Moreover, although rare, previous studies have illustrated the presence of intracardiac bubbles arising from portal vein gas in cirrhotic patients.^6^^,^^7^ While the portal vein was not directly visualized during our emergent TEE given the urgency of the situation, preoperative abdominal imaging studies had not shown any portal vein gas, thus making this etiology less likely. Once CO_2_ embolism was confirmed, immediate deflation of the pneumoperitoneum was recommended, and volume expansion with crystalloids, ventilation with high FiO_2_, and rotation of the surgical bed into Trendelenburg positioning were initiated. Over the next 5 minutes, hemodynamics improved significantly (SpO_2_ = 98%, systolic blood pressure = 100, diastolic blood pressure = 60), and no additional gas was visualized in the heart (Figures 3 and 4, Video 3).

Given that the patient was now stable, and the source of their cardiovascular collapse had been correctly elucidated, we recommended that the surgical team convert to open, thus avoiding the risk of further gas embolization. Our surgical colleagues agreed, and the remainder of the procedure was completed as a laparotomy, without complication. Subsequent TEE illustrated minimal remaining gas in the right atrium, right ventricle, right ventricular outflow tract/pulmonary artery, or left ventricle and normal biventricular systolic function (Video 4). A full resection was achieved, after which a central line was placed in the right internal jugular for further hemodynamic monitoring. At the end of the procedure, the patient was extubated, transferred to the postanesthesia care unit, and found to have no neurological deficits. During the postoperative period, no additional hemodynamic or ventilatory support was required. The patient was otherwise stable and transferred to a level 2 bed in the transplant unit, where they made an uncomplicated recovery. They were ultimately discharged from hospital on post-operative day 7 with no neurologic sequelae.

## Discussion

In current practice, CO_2_ is among the primary insufflation agents used for pneumoperitoneum. In rare instances, insufflated CO_2_ can become trapped in an injured vein, artery, or solid organ, ultimately precipitating CO_2_ embolism.^8^ This typically manifests clinically via a “gas lock” effect, whereby embolized CO_2_ molecules enter the right atrium and right ventricle, become entrapped, and obstruct outflow from the right heart, thus precipitating right and/or left heart failure, arrythmias, pulmonary hypertension, and ultimately, cardiovascular collapse.^9^ In such cases, prompt recognition is crucial. While TEE is an extremely sensitive tool for detecting mild to moderate-sized gas emboli and should be utilized when available, precordial Doppler and ETCO_2_ monitoring are suitable alternatives in most cases.^10^^,^^11^ Once CO_2_ embolism is confirmed, immediate discontinuation of pneumoperitoneum is indicated to prevent further gas entry. The patient may also be repositioned into a head-down (Trendelenburg) and left lateral decubitus (Durant’s) position to mitigate the risk of systemic embolization. Ventilation with high FiO_2_ will encourage CO_2_ washout and correct any V/Q mismatch. Vital signs should be continuously monitored, and cardiopulmonary resuscitation with inotropes and vasopressors should be initiated as needed to maintain end organ perfusion and cardiac output. If an injured vessel or organ is clearly identified as the source of systemic gas influx, prompt closure of this defect should prevent additional embolization. If the cause of the CO_2_ embolism is not readily apparent, surgery should be converted to open to avoid further insult.

In cases involving cardiovascular collapse of unknown origin while under general anesthesia, a systematic approach to delineating both the nature and cause of the decompensation is vital. Although the initial capnography, known patient history, and physical exam led us to first suspect bronchospasm as the cause of our patient’s acute decompensation, further investigations and a lack of improvement despite appropriate interventions directed us to consider other causes. While these steps may have ultimately delayed our diagnosis, they played a crucial part in excluding more common causes of postinduction desaturation. Importantly, after ruling out bronchospasm, anaphylaxis, circuit failure, and other iatrogenic causes, our repeat ABG, which showed a significant V/Q mismatch (ETCO_2_ = 39, pCO_2_ = 71), led us to consider causes of shunt. It was with this hypothesis in mind that an emergent TTE was performed, which confirmed our suspicions and illustrated massive CO_2_ embolism. By continually adapting to the evolving clinical situation and utilizing a systematic approach, we were able to discard our initial diagnoses of bronchospasm, and later anaphylaxis, and pursue additional causes based on the available data. Fortunately, this delay in diagnosis did not result in any adverse patient outcomes.

Moreover, this case also highlights the importance of the timely recognition and treatment of CO_2_ embolism in laparoscopic procedures. Although rare, clinically significant CO_2_ embolism carries a very poor prognosis and should thus always be kept in mind when witnessing hemodynamic and/or respiratory compromise of an unclear origin during laparoscopy. In skilled hands, TEE is an invaluable tool capable of detecting CO_2_ emboli as small as 0.1 mL/kg and should be promptly utilized if suspicion for CO_2_ embolism arises.^10^^,^^11^ If gas is visualized in the left side of the heart at any moment or a shunt-enabling intracardiac defect is present, the patient should be monitored closely in the postoperative period for any neurologic sequelae. If any predisposition or suspicion of paradoxical embolism is present, quick and simple interventions such as placing the patient into Durant’s positioning can help to immediately mitigate the risk of cerebral embolization. Crucially, given the rapid onset of cardiovascular collapse despite a lack of discernible vessel injury following an otherwise uncomplicated insufflation, our case also highlights the importance of close hemodynamic monitoring during initiation of pneumoperitoneum in laparoscopic procedures.

Despite our favorable patient outcome, this case should serve as a reminder of the massive hemodynamic alterations that can occur in cases of CO_2_ embolism. When witnessing cardiovascular collapse of an unknown origin during an otherwise uncomplicated procedure, it is essential that one keeps their differential broad and flexible—unnecessary cognitive rigidity may significantly delay diagnosis and worsen patient outcomes. Although exploring additional, more likely, causes for our patient’s deterioration may have confidently led us to correctly identifying the underlying process, a timelier ultrasonographic assessment would have surely resulted in prompter treatment. Unfortunately, the feasibility of implementing TEE prior to obvious cardiovascular collapse is a difficult question to ponder, as many centers suffer from limited resources and variable operator proficiency with more advanced intraoperative imaging techniques such as TEE. This dilemma ultimately warrants further discussion and may highlight the need for a broader use of less invasive and cheaper monitoring techniques such as precordial Doppler for routine surveillance against gas embolism in relatively higher-risk surgeries such as laparoscopic hepatectomy.^12^ In centers where TEE is readily available, our case suggests that it should be utilized early and with a very low threshold in laparoscopic procedures where unanticipated cardiorespiratory compromise is present to avoid missing a potentially devastating iatrogenic complication.

## Conclusion

Although exceedingly rare in comparison to more commonly encountered hemodynamic alterations such as hypotension and bradycardia, CO_2_ embolism remains a significant complication of pneumoperitoneum during laparoscopic surgery. In cases where the etiology remains elusive, a systematic approach to investigating potential causes and constant communication between the surgical and anesthesia teams is crucial to securing good patient outcomes. Importantly, even in the absence of definitive evidence for paradoxical embolism, all patients with clinically significant CO_2_ embolism should be closely monitored in the postoperative period to ensure no neurological deficits present. As witnessed by our case, the timely recognition and treatment of massive CO_2_ embolism during laparoscopy can lead to rapid reabsorption and favorable neurologic outcomes and negate the need for further circulatory or ventilatory support in the postoperative period.

## Ethics Statement

The authors declare that the work described has been carried out in accordance with The Code of Ethics of the World Medical Association (Declaration of Helsinki) for experiments involving humans.

## Consent Statement

Complete written informed consent was obtained from the patient (or appropriate parent, guardian, or power of attorney) for the publication of this study and accompanying images.

## Funding Statement

The authors declare that this report did not receive any specific grant from funding agencies in the public, commercial, or not-for-profit sectors.

## Disclosure Statement

The authors report no conflict of interest.

## Supplementary Data

Video 1 Two-dimensional TEE midesophageal 5-chamber view (0°), demonstrates a significant amount of echo-bright densities in the right heart and an empty left ventricle with near-systolic chamber obliteration during systole.

Video 2 Two-dimensional TEE, midesophageal short-axis view (80°), demonstrates the right ventricular inflow-outflow display with a significant amount of echo-bright densities in the right heart consistent with the massive air content.

Video 3 Two-dimensional TEE, midesophageal 4-chamber view (18°) approximately 5 minutes postconversion from laparoscopy to laparotomy, demonstrates a few remaining echo-bright densities in the right heart with adequate left ventricle filling and resolution of the previously noted leftward deviation of the atrial septum, suggesting normalization of previously elevated RA pressures.

Video 4 Two-dimensional TEE, midesophageal short-axis view (80°), demonstrates the right ventricular inflow-outflow display approximately 5 minutes postconversion to laparotomy with minimal amount of echo-bright densities remaining in the right heart. The atrial septum is now seen to deviate rightward.
